# Assessing progress under Health 2020 in the European Region of the World Health Organization

**DOI:** 10.1093/eurpub/ckaa091

**Published:** 2020-06-30

**Authors:** Mark R J Zuidberg, Amanda Shriwise, Lisanne M de Boer, Anne S Johansen

**Affiliations:** c1 Amsterdam UMC, Amsterdam, The Netherlands; c2 University of Bremen, Bremen, Germany; c3 University of Kansas, Lawrence, KS, USA; c4 Dutch Health and Youth Care Inspectorate, Utrecht, The Netherlands; c5 WHO Regional Office for Europe, Copenhagen, Denmark

## Abstract

**Background:**

Health 2020 is the regional health policy framework of the World Health Organization (WHO) Regional Office for Europe. The goals of Health 2020 are to improve health and well-being, reduce health inequalities and strengthen public health. To gain insight into the Health 2020 targets needing extra attention in coming years, we assessed progress under Health 2020 in the WHO European Region.

**Methods:**

Quantitative methods were used to assess progress in 50 out of 53 Member States of the WHO European Region in 2005, 2010 and 2015. The 16 quantitative Health 2020 indicators were rescaled from 1 to 100, with 1 indicating poor performance and 100 indicating good performance. The geometric mean of all 16 rescaled indicators was taken by Health 2020 target to compose a Health 2020 index.

**Results:**

The Health 2020 index (2015) ranged from 82.8 in Sweden to 30.0 in Turkmenistan. A clear east-west gradient was observed in the WHO European Region, with countries in western parts performing relatively better than countries in eastern parts. Indicators with the largest increase between 2005 and 2015 were premature mortality, mortality external causes, life expectancy and infant mortality. However, all quintiles showed a decline on overweight.

**Conclusions:**

The Health 2020 index gives a relative overview regarding the past and present performance on the Health 2020 policy framework of countries in the WHO European Region. Although improvements have been observed between 2005 and 2015, challenges remain to improve health for all in the context of the United Nations 2030 Agenda for Sustainable Development.

## Introduction

To address health determinants and tackle health disparities in Europe, the World Health Organization (WHO) Regional Office for Europe, together with its Member States, developed ‘Health 2020: a European policy framework and strategy for the 21st century’.^1^ This regional health policy framework was adopted in 2012 by all 53 Member States in the WHO European Region after an extensive two-year consultation process. The two strategic objectives of Health 2020 are ‘(i) to improve health for all and reduce health inequalities and (ii) to improve leadership and participatory governance for all’.^1^

Regional health policy frameworks present an opportunity for addressing cross-border threats to health and well-being, particularly those that are created or exacerbated by other integration, development and globalization processes,^2^ such as accession to the European Union.^3^ Furthermore, with 53 Member States and a population of more than 900 million, the WHO European Region is diverse in history, culture and development.^4^ Member States in the WHO European Region have been guided by Health 2020 in different ways and to different extents depending on their health and political priorities, institutional capacity and intervention capabilities. Indeed, by 2016, 98% of the Member States in the WHO European Region responding to an online questionnaire reported having a national policy addressing inequalities in health and/or social determinants of health, either as a stand-alone policy or incorporated into a national or subnational health policy or strategy.^5^

As 2020 approaches, it is important to assess the health progress of Member States in the WHO European Region under the Health 2020 policy framework. To this end, we developed a relative Health 2020 index for 2005, 2010 and 2015 of the Health 2020 targets and indicators for each country in line with similar approaches used to assess health and health-related targets in the context of the United Nations (UN) 2030 Agenda for Sustainable Development.^6^^,^^7^ Such indices can contribute to effective and sustainable health policy development by identifying regional health disparities, aide in setting priorities and focus future efforts to improve health and well-being within and between countries at the regional level. This is a necessary first step to ensure that regional health policy implementation is associated with improved health outcomes and to encourage cooperation and coherence in efforts to improve health and well-being for all throughout the WHO European Region in line with the UN 2030 Agenda for Sustainable Development.

## Methods

**Figure 1.**
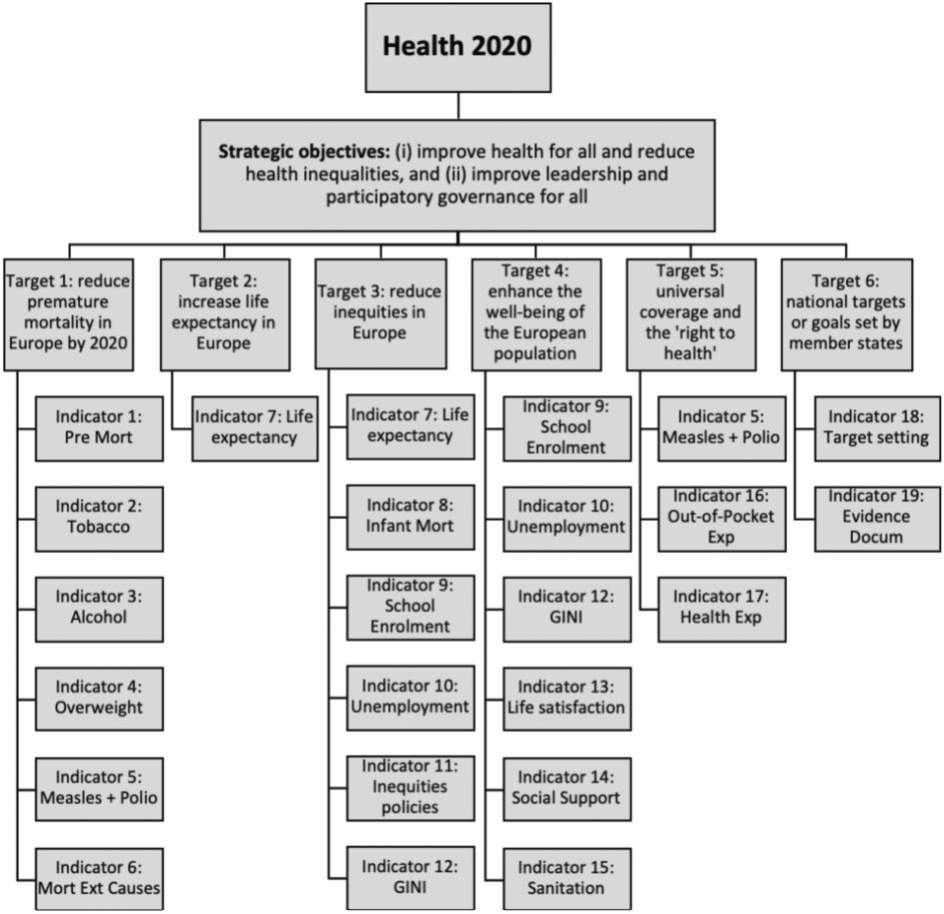
Health 2020 strategic objectives, targets and indicators. Displayed are the 2 strategic objectives, 6 targets and 19 indicators of Health 2020.

Figure 1 introduces the six targets and 19 indicators developed to monitor the health progress of Health 2020 (figure 1).^8^ During the development of the Health 2020 policy framework, the goals and targets were set first and afterwards the indicators were aligned accordingly. Detailed descriptions for the units of analysis for every indicator can be found in Supplementary appendix 1. The indicators measure the degree to which the targets have been achieved. Most indicators are used for one target, but some are used for two targets because they were aligned to more than one target during the development of the Health 2020 policy framework (figure 1).^1^^,^^8^

Similar to the construction of indices to measure progress on sustainable and human development,^7^^,^^9^ we constructed the Health 2020 index based on Health 2020s 16 quantitative indicators. This index was constructed for 50 out of 53 Member States of the WHO European Region. Liechtenstein is not a member of the WHO and therefore is not included; Andorra, Monaco and San Marino were excluded because data for more than six Health 2020 indicators is absent for these country cases. This cut-off point was used to ensure a good balance between the quality of the Health 2020 index on one hand and data availability on the other hand.

For each Member State, the index calculation consisted of two steps. First, the minimum and maximum values for 2005, 2010 and 2015 were identified to function as the goalposts for the indicator. From this, indicator values were transformed on a scale from 1 to 100, with a higher score representing better progress. The second step was to calculate the Health 2020 index by calculating the geometric mean from the Health 2020 targets, which were obtained from corresponding indicators. The geometric mean was used because data were normalized^10^ and it is a more sensitive tool for differences between values, with a high value on one indicator index partly compensating for a low value on another indicator index (partial substitutability).^11^ This in contrast to the arithmetic mean or harmonic mean, which have full substitutability or no substitutability properties, respectively. This means that the effect of any outlier is greatly dampened when using the geometric mean and that an *x*-percentage change in any value has the same effect as the same percentual change in another value.^11^

For the overall Health 2020 index, the geometric mean was calculated by target instead of by indicator, because it is assumed that the Health 2020 targets are equally important. Since indicators 7, 9, 10 and 11 (life expectancy, school enrolment, unemployment and GINI-coefficient, respectively) were aligned to more than one target during the development of the Health 2020 policy framework (figure 1),^1^^,^^8^ these indicators have relatively more impact on the overall Health 2020 index compared with other indicators. Similar to the construction of indices to measure progress on sustainable and human development,^7^^,^^9^ indicator indices of zero or close to zero were rounded up to one to avoid problems when calculating the geometric mean. For more information and details regarding data analysis and construction of the Health 2020 index, see Supplementary appendix 2.

Of the 19 indicators, 3 were excluded from this study (indicators 11, 18 and 19), because they were qualitative indicators (Supplementary appendix 1). For indicators two and 14 (tobacco and social support, respectively) data were only available for 2015. For indicators four and 10 (overweight and life satisfaction, respectively), data were only available for 2010 and 2015.

When data were unavailable for 2005, 2010 or 2015, data ± 2 years, but closest to 2005, 2010 or 2015, were used for that indicator (Supplementary appendix 2). This is displayed with an asterisk at the indicator index for that specific country. When data were completely unavailable, the indicator index of that specific indicator was excluded in the calculation of the corresponding Health 2020 target as well as from the overall Health 2020 index for the given country. In these cases, the calculation of the Health 2020 targets (and thus the Health 2020 index) is based on fewer indicators, making the Health 2020 index for these Member States less robust. For five countries, limited data were available to calculate the Health 2020 targets, resulting in the exclusion of these targets. Consequently, the Health 2020 index of Tajikistan, Azerbaijan, Albania, Montenegro and Turkmenistan is based on three targets only instead of five. These countries also are reported to have one of the lowest quality civil registration and vital statistics systems in the WHO European Region.^12^ Since the Health 2020 index is only as valid as the data used to calculate it, the results for these countries should be interpreted with care.

Finally, to highlight the differences within the WHO European Region and in line with similar efforts to create indices of health and health-related targets in the context of the UN 2030 Agenda for Sustainable Development,^7^ the 50 countries of the WHO European Region were divided into five quintiles based on the Health 2020 index for 2015: high Health 2020 index (2015) (Health 2020 index ≥75.8), upper middle Health 2020 index (2015) (Health 2020 index between 66.6 and 75.3), middle Health 2020 index (2015) (Health 2020 index between 54.7 and 66.1), lower middle Health 2020 index (2015) (Health 2020 index between 50.2 and 53.9) and low Health 2020 index (2015) (Health 2020 index ≤49.7).

## Results

**Figure 2.**
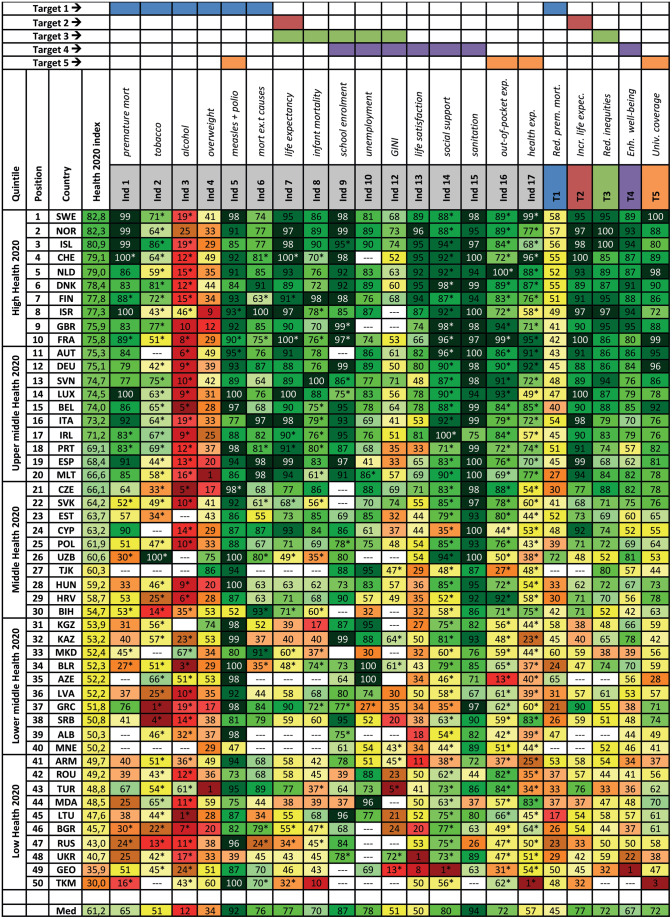
Health 2020 index (2015). Displayed is the Health 2020 index for 2015. Countries are ranked on the performance of the Health 2020 index (2015) from highest to lowest. Definitions of the Health 2020 indicators and targets can be found in Supplementary appendix 1. Indicators 11, 18 and 19 are qualitative and not included in this study. *Data ± 2 years was used for this indicator index. Med = median. For more information regarding the construction of the Health 2020 index, see Supplementary appendix 2.

Figure 2 shows the Health 2020 index for 2015 (for the Health 2020 indices for 2005 and 2010, see Supplementary appendix 3). The median Health 2020 index is 62.6. The three countries with the highest Health 2020 index (2015) are Sweden (82.8), Norway (82.3) and Iceland (80.9), and the three countries with the lowest Health 2020 index are Ukraine (40.7), Georgia (35.9) and Turkmenistan (30.0). Regarding Health 2020 target one (reduce premature mortality), Uzbekistan has the best performance with a target index of 72; Lithuania is performing the least well with a target index of 17 (figure 2). France, Switzerland and Luxemburg are performing the best on Health 2020 target two (increase life expectancy), with a score of 100. In contrast, Turkmenistan has a score of 32, performing the least well on this target (figure 2). For target three (reduce inequities), both Iceland and Norway have a target index of 100, whereas Georgia has the lowest target index with a score of 32. The country with the highest score on Health 2020 target four (enhance well-being) is the UK with 95, while Georgia has the lowest score for this target with a score of one. Finally, Sweden has the highest Health 2020 target index for target five (universal coverage) and Turkmenistan the lowest index with a score of three (figure 2).

**Figure 3.**
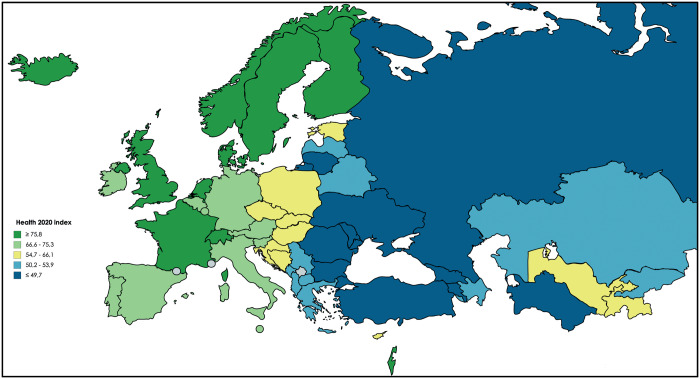
Map of Health 2020 index (2015). Displayed are the countries of the WHO European Region divided in quintiles based on the performance of the Health 2020 index (2015). Dark green = high Health 2020 quintile (2015), light green = upper middle Health 2020 quintile (2015), yellow = middle Health 2020 quintile (2015), light blue = lower middle Health 2020 quintile (2015) and dark blue = low Health 2020 quintile (2015).

The Health 2020 index for 2015 also shows a clear east-west gradient, with countries in the western part of the WHO European Region having a higher index compared with countries in the eastern part of the region (figure 3). Countries in the highest quintile were highly clustered and located primarily in Scandinavia and north-western Europe, with Israel being an exception. Countries in the second highest quintile were located in the south and middle parts of Europe. The countries in the third quintile were mainly located in central Europe, with the exception of Tajikistan and Uzbekistan. The fourth quintile comprises some countries of the Balkans and Eastern Europe, along with Kazakhstan, Azerbaijan and Kyrgyzstan. Finally, countries in the far south-east of Europe, Eastern Europe, the Caucasus, Russia and Turkmenistan were primarily in the lowest quintile.

**Figure 4.**
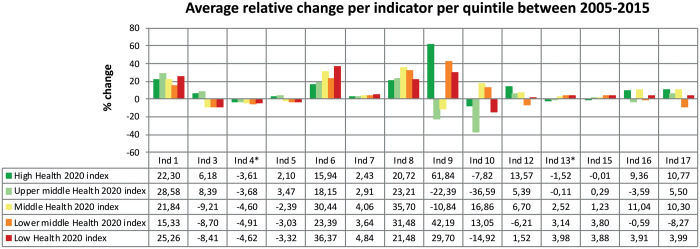
Average relative change per indicator, divided by quintile. Displayed is the average relative change per indicator between 2005 and 2015 per quintile. Positive values indicate improvement between 2005 and 2015 and negative values indicate a decline between 2005 and 2015. For indicators 2 and 14, no graph is shown because only data for 2015 was available. Indicator 11, 18 and 19 are qualitative and not included in this study. Definitions of the Health 2020 indicators can be found in Supplementary appendix 1. For the average absolute values for the years 2005, 2010 and 2015 per indicator per quintile, see Supplementary appendix 4. * = relative change between 2010 and 2015. Dark green = high Health 2020 quintile (2015), light green = upper middle Health 2020 quintile (2015), yellow = middle Health 2020 quintile (2015), orange = lower middle Health 2020 quintile (2015) and red = low Health 2020 quintile (2015).

As shown in figure 4, all quintiles showed improvements on indicators one (premature mortality), six (mortality external causes), seven (life expectancy) and eight (infant mortality) from 2005 to 2015. On the contrary, all quintiles showed a decline in progress on indicator 4 (overweight). Varied results can be observed in indicators nine and ten (school enrolment and unemployment), in which some quintiles show substantial improvements, whereas others show a notable decline between 2005 and 2015. For average absolute values for the years 2005, 2010 and 2015 per indicator and quintile, see Supplementary appendix 4.

## Discussion

To assess health progress in the WHO European Region, an index for each indicator, target and for the framework as a whole was calculated based on the 16 quantitative indicators of Health 2020 (figure 1). The Health 2020 index suggests a wide range of health outcomes across the region, ranging from 82.8 in Sweden to 30.0 in Turkmenistan. Furthermore, our Health 2020 index reveals a clear east-west gradient between Member States, with countries in the western part of the WHO European Region performing relatively better on Health 2020 than countries in the eastern part. A previous study on the Social Determinants of Health found a similar gradient, suggesting a need to identify and address the underlying root causes of the disparities in health and well-being both within and between countries.^4^ Taken together, this indicates that health policies can shape the health status of people throughout the WHO European Region, and that health is indeed a political choice.

This analysis also shows notable improvements from 2005 to 2015, since all quintiles improved on the indicators premature mortality, mortality external causes, life expectancy and infant mortality. In contrast, Member States across all quintiles have failed to make improvements on the indicator overweight and have instead regressed between 2005 and 2015. Without improvement in policy development and implementation as well as evidence-based interventions, Member States in the European region are at risk of continuing to decline in this area. A look beyond Europe shows that they are not alone. In the past 33 years, not a single country in the world has managed to decrease its overweight rates.^13^ If not effectively addressed, health risks associated with being overweight are expected to become even more pressing health policy issues in the future.

Between quintiles, mixed findings for the indicators school enrolment and unemployment are seen, indicating large disparities in the WHO European Region between 2005 and 2015. As reported in a number of studies, austerity measures in response to economic and financial crises may have affected both of these indicators.^14^^,^^15^ In particular, Greece’s health progress under Health 2020 has been set back in relative terms; its position declined from a ranking of 16 in 2005–37 in 2015.^15^

Although the Health 2020 index is a relative index, it gives a good overview of which country performs well on what indicator. In this way, it increased transparency and accountability and indicates which Health 2020 priority areas need extra attention in the coming years. While this analysis is unable to systematically account for or measure the impact of national and sub-national health policies, strategies and plans, a couple of examples illustrate the challenges inherent in health reform across Member States. For example, Turkey has made excellent progress on measles and polio vaccination as result of its Health Transformation Program (2003–13),^16^ but its rates of overweight are the highest among the Member States of the WHO European Region. This is not what observers may have hoped, considering the rigorous health reforms of Turkey in prior years. In contrast, progress of Uzbekistan under Health 2020 has been relatively good, with it having the best performance on tobacco use, measles and polio vaccination grades and sanitation levels in the WHO European Region. Despite this variation, 93% of the Member States in the WHO European Region report they have national health policies aligned with Health 2020,^5^ indicating mixed health outcomes in the European region while having the aspiration to achieve similar targets and indicators under Health 2020. Thus, in the coming years, Member States should focus on health policy effectiveness rather than health policy alignment with Health 2020.

Our analysis provides clear insight in the current status and progress made under Health 2020 in the WHO European Region and in this way can contribute to achieving improved health outcomes, effective and sustainable regional health policy development and the achievement of the sustainable development goals (SDGs) in the WHO European Region. Health 2020 was introduced before the adoption of the UN 2030 Agenda for Sustainable Development and its accompanying 17 goals and 169 targets. Goal 3 of this agenda is focused on health, and it specifies 13 targets to be achieved within the health sector, as well as number of health-related targets beyond goal 3 alone.^7^ Like Health 2020, efforts to improve health in the context of the 2030 Agenda have stressed that health impacts and is also impacted by policymaking and implementation efforts across *all* of the SDGs^17–19^; in this way, the forward thinking approach of Health 2020 gave the European region a head start in working across sectors, agencies and a growing number of stakeholders to improve health and well-being.^17^^,^^18^

At the request of Member States, the WHO Regional Office for Europe decided to adopt a Joint Monitoring Framework to harmonize indicators across Health 2020, the SDGs and the Global Action Plan for the Prevention and Control of Noncommunicable Diseases.^20^^,^^21^ The Joint Monitoring Framework aims to improve interoperability of health data while decreasing the burden of reporting for Member States. The quality of civil registration and vital statistics systems varies widely throughout the world and the WHO European Region.^12^ Member States with lower quality registration and statistical systems also have fewer health data available, highlighting the need to improve these systems in order to enhance health monitoring and evaluation.

The WHO Regional Office for Europe continues to monitor health trends beyond those covered within the SDGs. To this end, WHO has identified eight non-SDG indicators to include alongside 38 SDG indicators in the impact framework accompanying its 13th General Programme of Work, 2019–23. The eight indicators are in the areas of antimicrobial resistance (infections due to AMR; antibiotic consumption); polio; risk factors for communicable diseases (obesity; blood pressure; trans-fats) and emergency-related factors (vaccination for emergencies, essential health services for vulnerable populations).^22^ Regional health policy frameworks play a critical role in tailoring global frameworks to regional specificities, and it will be important to consider these eight targets in any future iterations of WHO’s regional health policy framework.

Given their role in providing technical support tailored to the needs to Member States throughout the European Region and the increasingly complex policy context in which this takes place, it will be interesting to see how the WHO Regional Office for Europe approaches regional health policy coordination beyond Health 2020, but one would hope that fighting the growing epidemic of overweight and obesity will be a high priority.

## Supplementary data

Supplementary data are available at *EURPUB* online.

## Funding

No funding was used for this article.

*Conflicts of interest*: Non declared.

**Key points**

- Sweden is relatively performing the best on Health 2020, whereas Turkmenistan is relatively performing the worst on Health 2020.
- Health 2020 performance differs between the eastern and western part of the World Health Organization (WHO) European Region.
- Between 2005 and 2015, all quintiles improved on the indicators premature mortality, mortality external causes, life expectancy and infant mortality and showed a decline on overweight.
- This analysis can contribute to effective and sustainable regional health policy development and the achievement of the sustainable development goals in the WHO European Region.

## Supplementary Material

ckaa091_supplementary_data
