# Supplementary material for: Assessing progress under Health 2020 in the European Region of the World Health Organization
Source: Eur J Public Health. 2020 Jun 30;30(6):1072–7. doi: 10.1093/eurpub/ckaa091 (PMC7733045; doi:10.1093/eurpub/ckaa091)
Supplement: ckaa091_supplementary_data [file ckaa091_supplementary_data.zip › ejph-2019-08-om-0701-File006.pdf]

Supplementary appendix 1

	Indicator short	Number indicator	Indicator definition	Indicator unit	Positive negative indicator	Source
**Target 1:** Reduce premature mortality in Europe by 2020						
**1.1.a**	Pre Mort	1	Age-standardized overall premature mortality rate for 4 major noncommunicable diseases (cardiovascular disease, cancer, diabetes mellitus, chronic respiratory disease)	Deaths per 100.000	Negative	Health 2020 database
**1.1.b**	Tobacco	2	Age-standardized prevalence of current tobacco use among people aged 18 years and over	%	Negative	Health 2020 database
**1.1.c**	Alcohol	3	Total per capita alcohol consumption among people aged 15 years and over within a calendar year	Litres per capita	Negative	Health 2020 database
**1.1.d**	Overweight	4	Age-standardized prevalence of overweight in people aged 18 years and over (defined as a BMI > 25 kg/m^2)	%	Negative	Health 2020 database

**1.2.a**	Measles + Polio	5	Percentage of children vaccinated against measles and polio	%	Positive	Health 2020 database
**1.3.a**	Mort Ext Causes	6	Age-standardized mortality rates from all external causes and injuries	Deaths per 100.000	Negative	Health 2020 database
**Target 2:** Increase life expectancy in Europe						
**2.1**	Life expectancy	7	Life expectancy at birth	Years	Positive	Health 2020 database
**Target 3:** Reduce inequities in health in Europe						
**3.1.a**	Infant Mort	8	Infant mortality per 1.000 life births	Deaths per 1.000 births	Negative	Health 2020 database
**3.1.b**	Life expectancy	7	Life expectancy at birth	Years	Positive	Health 2020 database
**3.1.c**	School enrolment	9	Proportion of children of official primary school age not enrolled	%	Negative	Health for All database
**3.1.d**	Unemploy ment	10	Unemployment rate	%	Negative	Health 2020 database
**3.1.e**	Inequities Policies	11	National and/or subnational policy	n.a.	n.a.	n.a.

			addressing the reduction of health inequities established and documented			
**3.1.f**	GINI	12	GINI coefficient (income distribution)	Score between 0-100	Negative	Health for All database
**Target 4:** Enhance the well-being of the European population						
**4.1.a**	Life satisfaction	13	Life satisfaction	Score between 0-10	Positive	World Happiness Report
**4.1.b**	Social Support	14	Availability of social support	%	Positive	Health for All database
**4.1.c**	Sanitation	15	Percentage of population with improved sanitation facilities	%	Positive	Health 2020 database
**4.1.d**	GINI	12	GINI coefficient (income distribution)	Score between 0-100	Negative	Health for All database
**4.1.e**	Unemployment	10	Unemployment rate	%	Negative	Health 2020 database

**4.1.f**	School enrolment	9	Proportion of children of official primary school age not enrolled	%	Negative	Health for All database
**Target 5:** Universal coverage and the "right to health"						
**5.1.a**	Out-of-Pocket Exp	16	Private household out-of-pocket expenditure as a proportion of total health expenditure	%	Negative	Health 2020 database
**5.1.b**	Measles + Polio	5	Percentage of children vaccinated against measles and polio	%	Positive	Health 2020 database
**5.1.c**	Health Exp	17	Total expenditure on health (as a percentage of GDP)	%	Positive	Health 2020 database
**Target 6:** National targets/goals set by Member States						
**6.1.a**	Target setting	18	Establishment of process for target-setting documented	n.a.	n.a.	n.a.
**6.1.b**	Evidence Documenting	19	Evidence documenting: (a) establishment of national policies aligned with Health 2020; (b) implementation plan; (c) accountability mechanism	n.a.	n.a.	n.a.
