# Supplementary material for: Assessing progress under Health 2020 in the European Region of the World Health Organization
Source: Eur J Public Health. 2020 Jun 30;30(6):1072–7. doi: 10.1093/eurpub/ckaa091 (PMC7733045; doi:10.1093/eurpub/ckaa091)
Supplement: ckaa091_supplementary_data [file ckaa091_supplementary_data.zip › ejph-2019-08-om-0701-File007.pdf]

<u>Supplementary appendix 2</u>

Data collection

In September 2018, data for the indicators were retrieved from the Health 2020 database, except for indicators 9, 12, 13, and 14. For these indicators, no data was available on the Health 2020 database. Therefore, data for indicators 9, 12, and 14 were derived from the Health for All Database and for indicator 13 the World Happiness Report was used.

Data for the indicators were collected for the years 2005, 2010, and 2015. If no data was available for these years, data +/- 2 years but closest to 2005, 2010, or 2015 were used (orange table 1). If this 'old' (orange) data were used for one of the Health 2020 indices, this is displayed with an asterisk at the indicator index for that specific country.

If no data was available between 2003 and 2007 for the Health 2020 index (2005), between 2008 and 2012 for the Health 2020 index (2010), and between 2013 and 2017 for the Health 2020 index (2015) (Table 1), the indicator index of that specific indicator for the given country was excluded in the calculation of the Health 2020 index. This was displayed as empty space in the indicator index of that indicator.

2003	2004	2005	2006	2007
2008	2009	2010	2011	2012
2013	2014	2015	2016	2017

Table 1. Data range. *Displayed is the range for data collection. Orange data is referred to with an asterisk.*

Data analysis

The analysis of progress on the sixteen quantitative Health 2020 indicators was based on the methodology of the Human Development Index (HDI). This analysis consists of two steps. First, the minimum and maximum values between 2005, 2010, and 2015 were determined for every indicator, and an indicator index was calculated. Second, the geometric mean of all indicators was calculated by target for every country.

The first step for the analysis of the performance on the Health 2020 indicators was to identify the dimensions for each indicator and to calculate the indicator index. For this, it was important that minimum and maximum values were set, because they function as the 'goalposts' for that indicator. The minimum and maximum values were the lowest and highest value observed for the given indicator between 2005, 2010, and 2015. Indicator indices of zero or close to zero were rounded up to one to avoid problems with the calculation of the geometric mean. In the formula below, the actual value was the value of a specific country for a given indicator. Having defined the 'goalposts' for an indicator, the formula for the indicator index was the following:

$$Indicator\ index = \frac{(actual\ value - minimum\ value)}{(maximum\ value - minimum\ value)}\ x\ 100$$

Some indicators are negative indicators (indicators 1, 2, 3, 4, 6, 8, 9, 10, 12, 16), in which a lower value corresponds to better progress. Other indicators are positive indicators (indicators 5, 7, 13, 14, 15, 17), in which a higher value corresponds to better progress. In other words, the lowest value was represented as the best performance for negative indicators, whereas the highest value was represented as best performance for positive indicators. Because of this difference, a different approach was used to calculate the indicator index for negative indicators. In that case, the indicator index was subtracted from 100 to get the actual indicator index.

To clarify the formula for the indicator index, an example is provided below. For example, if premature mortality for a given country is 600 deaths per 100.000, and the minimum value is 100, and the maximum value is 900, the indicator index for that indicator would be:

$$Indicator\ index = \frac{(600 - 100\ )}{(900 - 100\ )}\ x\ 100$$

$$Indicator\ index = \frac{500}{800}\ x\ 100 = 62.5$$

The calculation of the indicator index was executed for each indicator for each country.

The second step for the Health 2020 index was to calculate the geometric mean from all indicator indices by target for each country. The geometric mean is the mean of a set of values by multiplying them and then take the *n*th root of the answer of *n* numbers. The geometric mean was used because it is a more respectful tool for differences between indicator indices. In other words, it makes sure that a 1% decline in a specific indicator index has the same impact as a 1% decline in another indicator index. In light of this study, the geometric mean of the indicators by target represent the Health 2020 index.

The formula for the geometric mean is:

$$Geometric\ mean = \sqrt[5]{target\ 1 \times target\ 2 \times target\ 3 \times target\ 4 \times target\ 5}$$

Regarding the five Health 2020 targets, the formulas are:

$$Target\ 1 = \sqrt[6]{ind\ index_1 \times ind\ index_2 \times ind\ index_3 \times\ ind\ index_4 \times\ ind\ index_5 \times\ ind\ index_6}$$

$$Target\ 2 = \sqrt[1]{\ ind\ index_7}$$

$$Target\ 3 = \sqrt[5]{ind\ index_7 \times\ ind\ index_8 \times\ ind\ index_9 \times\ ind\ index_{10} \times ind\ index_{12}}$$

$$Target\ 4 = \sqrt[6]{ind\ index_9 \times ind\ index_{10} \times ind\ index_{12} \times ind\ index_{13} \times ind\ index_{14} \times ind\ index_{15}}$$

$$Target\ 5 = \sqrt[3]{ind\ index_5 \times ind\ index_{16} \times ind\ index_{17}}$$

By rewriting the formula above, the formula for the Health 2020 index is:

$$Health\ 2020\ index = (target\ 1 \times target\ 2 \times target\ 3 \times target\ 4 \times target\ 5)^{1/5}$$

To clarify the formula for the Health 2020 index, an example is provided. For example, if the target scores for the five targets are 43, 61, 28, 3, 89, the Health 2020 index is:

$$Health\ 2020\ index = (43 \times 61 \times 28 \times 3 \times 89)^{1/5}$$

$$Health\ 2020\ index = 19.609.548^{1/5} = 28.74$$

Finally, the Health 2020 index scores of all countries in the WHO European Region were presented from highest to lowest to provide an overview of the progress of countries regarding Health 2020.
