# Supplementary material for: Assessing progress under Health 2020 in the European Region of the World Health Organization
Source: Eur J Public Health. 2020 Jun 30;30(6):1072–7. doi: 10.1093/eurpub/ckaa091 (PMC7733045; doi:10.1093/eurpub/ckaa091)
Supplement: ckaa091_supplementary_data [file ckaa091_supplementary_data.zip › ejph-2019-08-om-0701-File008.pdf]

Supplementary appendix 3

Quintile	Position	Country	Health 2020 index	premature mort Ind 1	alcohol Ind 3	measles + polio Ind 5	mort ext. causes Ind 6	life expectancy Ind 7	infant mortality Ind 8	school enrolment Ind 9	unemployment Ind 10	GINI Ind 12	sanitation Ind 15	out-of-pocket exp. Ind 16	health exp. Ind 17	Red. Prem. Mort. T1	Incr. life expec. T2	Red. inequities T3	Enh. Well-being T4	Univ. coverage T5
Target 1 →				■	■	■	■									■				
Target 2 →								■									■			
Target 3 →								■	■	■	■	■						■		
Target 4 →										■	■	■	■						■	
Target 5 →						■								■	■					■
High Health 2020	1	ISL	77.5	85	20	85	79	92	88	87	94	53	97	84	72	58	92	90	82	82
	2	SWE	76.2	81	22	95	72	88	86	72	80	61	98	85	70	60	88	84	77	85
	3	NOR	73.5	80	23	80	70	86	78	91	89	42	95	86	68	56	86	81	76	79
	4	CHE	73.2	88	11	80	76	92	67	88	91	49*	100	67	88	48	92	82	80	79
	5	NLD	73.1	71	12	92	88	82	62	90	85	51	94	97	75	51	82	79	79	91
	6	ITA	72.7	80	15	98	85	89	73*	96	80	35	99	80	67	56	88	76	71	82
	7	MLT	72.6	74	23	79	91	81	55	---	88	64*	100	69	68	59	81	77	84	71
	8	GBR	71.6	68	7	71	87	80	61	100	88	---	98	94	62	39	80	90	100	75
	9	FRA	70.8	77	6	85	63	86	73	96	77	51	97	98	85	38	86	82	78	93
	10	DNK	69.8	65	8	88	73	76	66	77	88	65	99	89	77	42	75	81	83	87
Upper middle Health 2020	11	FIN	69.7	73	11	95	47	81	78	84	79	59	93	81	64	43	80	83	79	80
	12	ESP	69.2	78	7	94	82	86	71	98	76	41	100	79	61	44	86	78	74	78
	13	DEU	68.9	70	7	87	83	82	70	98	71	40*	98	89	85	42	81	75	72	90
	14	SVN	68.2	61	9	89	52	71	68	92	84	70	98	91	65	38	71	84	88	82
	15	LUX	66.9	26	7	95	75	85	87	86	89	46	94	92	59	31	85	84	76	82
	16	GRC	66.5	74	11	100*	79	80	71	82	74	31	92	62	73	50	80	68	63	77
	17	BEL	66.5	71	6	85	61	80	69	79	78	51	99	84	72	37	79	76	75	82
	18	AUT	65.5	73	6	57	67	82	68	---	86	55	100	84	85	33	82	78	79	74
	19	CYP	64.4	83	8	84	60	80	63	98	87	49	100	46	43	41	80	80	82	50
	20	IRL	62.4	70	4	72	75	80	70	99	89	36	72	86	52	33	80	77	68	67
Middle Health 2020	21	ISR	61.8	83	48	89	82	85	66	85	77	6	100	69	54	75	85	46	38	68
	22	CZE	60.8	46	4	94	58	64	75	---	80	62	98	93	49	29	63	75	79	76
	23	HRV	59.6	43	8	93	57	59	57	93	67	51*	93	89	49	34	59	68	74	74
	24	ALB	57.3	70	28	95	67	67	45	45*	63	48	66	39	41	60	67	53	50	48
	25	PRT	56.9	69*	4	86	67*	76*	70*	---	81	19	94	78	79	33	75	54	47	83
	26	SVK	56.7	35	9	99	57	53	49	---	57	71*	97	78	50	35	52	59	73	72
	27	POL	53.6	40	13	99	52	58	53	68	54	34	75	73	42	38	57	52	51	65
	28	MNE	52.5	36	27*	79*	76	52	39	87	19	49	76	69	64	48	52	42	44	70
	29	SRB	52.5	27	12	95	67	45	45	98	42	37	91	68	67	36	44	50	58	76
	30	HUN	50.9	22	5	100	48	46	54	77	85*	31	95	74	62	23	45	56	64	78
Lower middle Health 2020	31	ROU	50.2	26	18	95	55	41	24	86	79*	51	35	83	34	38	41	51	56	63
	32	BIH	49.3	---	32	85	---	---	---	---	17*	35*	86	51	65	---	---	---	29	63
	33	TJK	48.4	32*	96	92	80	50	32	84	---	37*	79	5	39	70	49	46	60	15
	34	BGR	47.3	22	10	94	66	43	36	61	74	29*	62	58	50	32	43	45	49	63
	35	EST	44.8	33	1	93	26	46	59	76	80	37	92	80	30	13	46	59	66	58
	36	UZB	44.3	16	56	100	63	31	24	82*	---	31*	93	39	31	48	31	33	59	43
	37	ARM	43.8	34*	33	86	76*	46*	28*	22	79	28	72	27	32	52	46	32	37	34
	38	LVA	40.0	77	12	100	18	32	47	---	74	15	56	54	43	33	32	32	32	59
	39	GEO	39.9	36	31	69	88	52	14	43	64	12	80	7	66	50	51	24	32	22
	40	MKD	39.6	36	56	95	84	50	29	---	1	---	73	55	59	64	50	1	---	66
Low Health 2020	41	LTU	39.2	19	6	91	11	36	49	44	79	31	69	66	38	14	35	44	47	58
	42	TUR	39.2	---	64	80	---	---	30*	86	76	3	75	77	34	---	---	22	27	56
	43	BLR	37.5	6*	9	99	9	21	53	39	97	59	86	81	49	11	20	47	64	73
	44	MDA	34.6	11	9	98	26	15	30	59	96	---	30	49	71	19	14	36	51	69
	45	KGZ	32.4	11	44	99	35	14	1	66	93	---	79	34	38	34	14	8	80	45
	46	UKR	31.4	5	19	92	16	12	38	35	82	54	87	58	44	15	11	33	57	59
	47	TKM	31.4	5	44	100	60	22	36	---	---	---	1	52	15	31	21	---	---	35
	48	AZE	31.2	23*	79	96	90	43*	31*	1	98	100	30	1	58	64	42	20	12	4
	49	KAZ	21.6	1	21	100	12	3	23	92	79	52	92	58	21	8	2	25	77	43
	50	RUS	11.0	1	8	99	1	1	34	55*	82	8	26	66	32	1	1	7	21	56
			56.8	45	11	93	67	61	55	84	80	46	92	74	59	38	61	57	67	71

Health 2020 index (2005). *Displayed is the Health 2020 index for 2005. Countries are ranked on the performance of the Health 2020 index (2005) from highest to lowest. Definitions of the Health 2020 indicators and targets can be found in supplementary appendix 1. Indicators 11, 18, and 19 are qualitative and not included in this study. For indicators two, four, thirteen, and fourteen (tobacco, overweight, life satisfaction, and social support), no data was available in 2005. * = data +- 2 years was used for this indicator index. Med = median. For more information regarding the construction of the Health 2020 index, see supplementary appendix 2.*

Quintile	Position	Country	Health 2020 index	premature mort. Ind 1	alcohol Ind 3	overweight Ind 4	measles + polio Ind 5	mort. ext. causes Ind 6	life expectancy Ind 7	infant mortality Ind 8	school enrolment Ind 9	unemployment Ind 10	GINI Ind 12	life satisfaction Ind 13	sanitation Ind 15	out-of-pocket exp. Ind 16	health exp. Ind 17	red. prem. mort. T1	incr. life expec. T2	red. inequities T3	enh. Well-being T4	univ. coverage T5
Target 1 →				■	■	■	■	■										■				
Target 2 →									■										■			
Target 3 →									■	■	■	■	■							■		
Target 4 →											■	■	■	■	■						■	
Target 5 →							■									■	■					■
High Health 2020	1	SWE	80.5	92	20	49	96	77	93	85	96	78	72	94	98	86	74	58	93	94	90	88
	2	NOR	79,5	87	22	42	86	72	91	85	94	91	74	99	95	88	72	54	91	97	94	84
	3	NLD	79.3	78	13	45	94	90	90	71	99	88	67	95	94	100	84	52	90	91	91	97
	4	CHE	78.1	95	11	57	88	81	98	71	94	89	52	99	100	73	90	53	98	87	87	86
	5	ISL	77.2	94	16	36	89*	73	94	89	92	81	66	91	97	83	68	51	94	93	87	81
	6	ITA	74.9	88	21	41	87	92	97	76	95	79	44	55	99	81	74	57	97	82	70	82
	7	DEU	74.4	76	8	46	92	86	87	75	98	82	53	73	98	89	92	46	87	85	80	95
	8	FIN	74.0	79	12	41	100*	53	86	89	87	79	67	92	94	82	70	45	86	90	85	85
	9	GBR	73.6	77	9	22	82	90	88	66	96	80	---	78	98	94	75	39	88	91	91	86
	10	BEL	73.3	77	11	33	99	64	85	73	92	79	63	81	99	84	81	43	85	86	83	91
Upper middle Health 2020	11	DNK	72.9	75	11	51	73	84	81	75	77	85	62	100	99	90	90	47	81	83	85	86
	12	SVN	72.5	72	11	51	92	57	84	85	83	82	73	56	98	91	70	44	84	90	78	86
	13	LUX	72.1	85	8	40	93	70	92	83	71	89	58	83	94	94	56	43	92	85	79	80
	14	FRA	71.3	83	7	37	66	68	94	74	95	76	51	75	97	97	91	38	94	84	77	86
	15	AUT	69.9	78	6	57	54*	74	89	70	---	88	57	91	100	85	91	39	88	81	84	75
	16	ESP	69.3	86	12	27	93	96	96	77	98	47	37	63	100	81	75	47	96	71	62	85
	17	CYP	69.2	93	8	37	86	84	95	81	96	85	50	67	100	48	52	44	95	87	78	56
	18	IRL	67.9	75*	7	36	84	77	88	71	98	64	48	84	74	83	67	40	88	78	71	78
	19	GRC	67.5	79	14	26	100*	88	88	71	91	67	39	39	96	69	71	47	87	73	59	80
	20	PRT	67.0	82	6	48	94	80	84	85	97	72	36	30	97	77	84	44	84	77	56	87
Middle Health 2020	21	CZE	64.2	54	5	24	98*	63	72	83	---	82	69	62	98	88	54	31	72	84	77	78
	22	TJK	62.4	---	100	100	89	---	---	---	88	96	47*	11	83	20	40	100	---	80	46	34
	23	MLT	62.0	82	17	7	42	92	92	57	89*	83	55	53	100	64	63	31	92	80	74	51
	24	SVK	61.3	44	11	50	100	62	61	57	---	62	66	54	97	79	65	41	60	64	66	81
	25	ALB	60.5	81*	28	51	100	82*	82*	58*	44	65*	50*	42	76	38	33	63	82	61	50	44
	26	HRV	59.7	50	6	39	94	59	67	66	66	69	44	45	92	89	62	35	67	65	58	82
	27	POL	59.6	46	10	42	95	57	65	62	80	68	46	50	85	78	48	38	65	67	61	71
	28	ISR	58.8	93	46	16	92	90	95	72	84	83	3	89	100	75	53	56	95	39	37	71
	29	HUN	57.2	28	9	31	100	56	56	59	90	71	72	22	95	73	58	32	55	74	60	75
	30	MNE	54.6	45*	22	39	82	77	61*	56*	50*	48	54	35	83	56	49	47	60	54	47	57
Lower middle Health 2020	31	BIH	54.6	51*	32	64	82	87*	66*	56*	---	27	36*	22	86	70	75	59	66	42	29	76
	32	UZB	54.0	26	45	88	99	78	46	34	67	---	---	44	100	48	33	60	45	46	65	49
	33	SRB	53.6	33	12	47	86	74	54	51	66	47	52	22	91	60	80	40	53	54	45	74
	34	EST	52.8	46	1	49	89	43	63	76	94	56	45	39	93	83	42	21	63	69	58	66
	35	ROU	50.2	34	14	47	89	59	50	38	60	82	37	28	40	82	38	39	50	51	40	63
	36	ARM	47.2	33*	33	59	94	69*	51*	33	---	83	46	9	72	34	25	53	50	50	32	36
	37	LTU	46.8	32	5	34	92	24	48	62	96	53	24	39	75	72	51	23	47	52	46	68
	38	LVA	46.8	25	12	41	87	36	47	58	88	48	28	29	61	61	45	30	46	50	40	59
	39	MKD	45.6	39	61	43	94	87	58	47	---	14	---	16	75	59	48	61	57	29	15	62
	40	TUR	44.6	66	60	12	95	99	68	31	77	71	1	37	81	86	36	54	68	19	17	65
Low Health 2020	41	BGR	44.4	28	9	29	92	77	50	40	96	74	38	1	62	51	52	33	50	57	18	60
	42	KGZ	43.6	21	45	88	87	38	25	10	61	94	---	29	81	57	46	48	25	30	57	58
	43	GEO	43.0	78	20	64	81	98	56	34	100*	57	5	6	71	17	80	60	55	31	15	42
	44	BLR	41.9	16	2	37	100	17	31	69	46	99	56	41	85	81	35	14	31	58	59	64
	45	MDA	41.4	16	16	68	95	30	23	32	38	90	---	49	34	49	100	33	22	37	43	79
	46	KAZ	40.5	13	21	64	99	25	20	20	98	86	56	46	93	52	24	31	19	44	72	44
	47	AZE	37.7	---	53	66	95	---	---	---	5	99	44*	17	51	17	33	70	---	21	18	29
	48	RUS	34.0	14	9	45	98	17	23	47	77*	81	10	40	26	51	48	21	22	32	28	59
	49	TKM	32.5	18	47	75	96	78	36	11	---	---	---	44	---	57	1	54	35	---	---	4
	50	UKR	28.7	17	16	41	1	37	30	41	65	79	70	29	88	54	58	9	29	54	59	1
		Med	60.1	69	12	42	92	74	70	66	89	79	51	45	94	76	60	44	70	69	60	75

Health 2020 index (2010). *Displayed is the Health 2020 index for 2010. Countries are ranked on the performance of the Health 2020 index (2010) from highest to lowest. Definitions of the Health 2020 indicators and targets can be found in supplementary appendix 1. Indicators 11, 18, and 19 are qualitative and not included in this study. For indicators two and fourteen (tobacco and social support), no data was available in 2010. * = data +- 2 years was used for this indicator index. Med = median. For more information regarding the construction of the Health 2020 index, see supplementary appendix 2.*
