# Supplementary material for: Assessing progress under Health 2020 in the European Region of the World Health Organization
Source: Eur J Public Health. 2020 Jun 30;30(6):1072–7. doi: 10.1093/eurpub/ckaa091 (PMC7733045; doi:10.1093/eurpub/ckaa091)
Supplement: ckaa091_supplementary_data [file ckaa091_supplementary_data.zip › ejph-2019-08-om-0701-File009.pdf]

Supplementary appendix 4

For some indicators, a decline in absolute value represents a relative improvement and thus a positive relative value (green). For example, indicator 1 is premature mortality, so a decline in absolute value represents improvement and thus a positive relative value. These indicators are called negative indicators (a higher value represents bad health outcomes). On the contrary, there are also positive indictors, in which a higher value represents good health outcomes. An example is indicator five, measles and polio vaccination.

Ind		Quintile	2005	2010	2015	% Improvement / decline
1	**Premature mortality**					
		High Health 2020	265.84	232.61	206.55	22.30 %
		Upper middle Health 2020	321.84	252.00	229.87	28.58 %
		Middle Health 2020	514.32	437.91	401.99	21.84 %
		Lower middle Health 2020	567.62	526.56	480.63	15.33 %
		Low Health 2020	717.47	566.33	536.22	25.26 %
3	**Alcohol**					
		High Health 2020	8.77	8.66	8.22	6.18 %
		Upper middle Health 2020	11.29	10.40	10.35	8.39 %
		Middle Health 2020	9.17	9.02	10.01	-9.21 %
		Lower middle Health 2020	6.33	6.77	6.88	-8.70 %
		Low Health 2020	7.32	7.58	7.94	-8.41 %
4	**Overweight**					
		High Health 2020	-	56.26	58.29	-3.61 %
		Upper middle Health 2020	-	56.51	58.59	-3.68 %
		Middle Health 2020	-	53.05	55.49	-4.60 %
		Lower middle Health 2020	-	53.58	56.21	-4.91 %

		Low Health 2020	-	54.31	56.82	-4.62 %
5	**Measles + polio**					
		High Health 2020	93.05	93.35	95.00	2.10 %
		Upper middle Health 2020	92.25	91.75	95.45	3.47 %
		Middle Health 2020	96.35	96.15	94.05	-2.39 %
		Lower middle Health 2020	97.25	96.05	94.30	-3.03 %
		Low Health 2020	94.90	91.90	91.75	-3.32 %
6	**Mortality ext. causes**					
		High Health 2020	39.37	35.85	33.09	15.94 %
		Upper middle Health 2020	39.23	35.22	32.11	18.15 %
		Middle Health 2020	60.43	48.20	42.03	30.44 %
		Lower middle Health 2020	76.10	65.64	58.30	23.39 %
		Low Health 2020	93.00	65.71	59.17	36.37 %
7	**Life expectancy**					
		High Health 2020	80.19	81.39	82.14	2.43 %
		Upper middle Health 2020	79.47	81.05	81.78	2.91 %
		Middle Health 2020	74.51	76.62	77.53	4.06 %
		Lower middle Health 2020	72.26	74.14	74.89	3.64 %
		Low Health 2020	70.29	72.63	73.69	4.84 %
8	**Infant mortality**					
		High Health 2020	3.75	3.22	2.98	20.72 %
		Upper middle Health 2020	3.98	3.46	3.06	23.21 %
		Middle Health 2020	7.32	5.12	4.70	35.70 %
		Lower middle Health 2020	11.30	8.64	7.74	31.48 %

		Low Health 2020	12.15	10.90	9.54	21.48 %
9	**School enrolment**					
		High Health 2020	2.11	1.36	0.80	61.84 %
		Upper middle Health 2020	1.19	1.42	1.45	-22.39 %
		Middle Health 2020	2.81	2.72	3.11	-10.84 %
		Lower middle Health 2020	5.85	6.26	3.38	42.19 %
		Low Health 2020	7.27	3.85	5.11	29.70 %
10	**Unemploy-ment**					
		High Health 2020	6.04	6.76	6.51	-7.82 %
		Upper middle Health 2020	7.04	9.14	9.62	-36.59 %
		Middle Health 2020	13.11	12.14	10.90	16.86 %
		Lower middle Health 2020	13.75	12.73	11.96	13.05 %
		Low Health 2020	8.27	9.88	9.50	-14.92 %
12	**GINI**					
		High Health 2020	30.49	28.15	26.35	13.57 %
		Upper middle Health 2020	31.22	29.41	29.54	5.39 %
		Middle Health 2020	31.76	29.29	29.63	6.70 %
		Lower middle Health 2020	30.32	30.78	32.21	-6.21 %
		Low Health 2020	36.27	35.77	35.72	1.52 %
13	**Life satisfaction**					
		High Health 2020	-	7.37	7.26	-1.52 %
		Upper middle Health 2020	-	6.46	6.45	-0.11 %
		Middle Health 2020	-	5.52	5.66	2.52 %
		Lower middle Health 2020	-	5.15	5.31	3.14 %
		Low Health 2020	-	5.02	5.22	3.98 %

**15**	**Sanitation**					
		High Health 2020	98.90	98.90	98.89	-0.01 %
		Upper middle Health 2020	98.22	98.39	98.50	0.29 %
		Middle Health 2020	96.52	97.30	97.71	1.23 %
		Lower middle Health 2020	90.33	92.22	93.76	3.80 %
		Low Health 2020	82.65	85.42	85.86	3.88 %
**16**	**Out-of-pocket exp.**					
		High Health 2020	16.75	15.43	15.18	9.36 %
		Upper middle Health 2020	18.19	18.42	18.84	-3.59 %
		Middle Health 2020	33.85	30.35	30.11	11.04 %
		Lower middle Health 2020	42.22	40.11	42.47	-0.59 %
		Low Health 2020	40.52	39.67	38.94	3.91 %
**17**	**Health exp.**					
		High Health 2020	9.21	9.73	10.20	10.77 %
		Upper middle Health 2020	8.97	9.58	9.46	5.50 %
		Middle Health 2020	6.62	7.34	7.31	10.30 %
		Lower middle Health 2020	7.16	6.69	6.57	-8.27 %
		Low Health 2020	6.20	6.91	6.44	3.99 %
